# Intervening pyruvate carboxylase stunts tumor growth by strengthening anti-tumor actions of tumor-associated macrophages

**DOI:** 10.1038/s41392-021-00807-w

**Published:** 2022-02-02

**Authors:** Yuxin Shu, Nanfei Yang, Nan Cheng, Zhengyun Zou, Wenlong Zhang, Yuncheng Bei, Qian Shi, Menghao Qin, Wei-Guo Zhu, Pingping Shen

**Affiliations:** 1 State Key Laboratory of Pharmaceutical Biotechnology and The Comprehensive Cancer Center, Nanjing Drum Tower Hospital, The Affiliated Hospital of Nanjing University Medical School, School of Life Sciences, Nanjing University, Nanjing, 210023 PR China; 2 Guangdong Key Laboratory of Genome Instability and Human Disease, Shenzhen University International Cancer Center, Department of Biochemistry and Molecular Biology, Shenzhen University, Shenzhen, 518060 PR China; 3 Department of Cellular and Integrative Physiology, The University of Texas Health Science Center at San Antonio, 7703 Floyd Curl Drive, San Antonio, TX 78229-3904 USA

**Keywords:** Tumour immunology, Skin cancer

**Dear Editor**,

Tumor-associated macrophages (TAMs) are critical pro-tumor immunocytes and depletion of TAMs has been exploited for cancer therapy.^1^ However, the phenotypes and functions of TAMs are plastic, TAMs can also be effector cells by engulfing tumor cells and recruiting cytotoxic T cells, thus shaping the actions of TAMs is more scientific rational than depleting them indiscriminately.^2^ To promote the entry of reorienting TAMs into the clinical treatments of tumors, it is essential to explore the underline mechanisms controlling the anti- and pro-tumor activities of TAMs.

As the seminal step in gluconeogenesis, pyruvate carboxylase (PCB) plays a dominant role in controlling gluconeogenesis.^3^ Although the correlation between tumor prosperity and the canonical functions of PCB in metabolism is gradually being revealed, it is still unknown whether and how PCB regulates the functions of immune cells, especially TAMs, in the tumor microenvironment. Our study aimed to investigate the role of PCB in shaping TAMs and evaluated the potential value of intervening PCB in treating cancer.

We firstly detected PCB expression and activity in TAMs and found both were significantly decreased in TAMs compared with spleen macrophages (Fig. 1a, Supplementary Fig. 1a–c) and that the reduction of PCB persisted throughout tumor progression (Fig. 1b, Supplementary Fig. 1d, e). Moreover, PCB reduction in TAMs was also observed in human melanomas (Fig. 1c, Supplementary Fig. 1f). Notably, unlike PCK1 and G6PC, two other key enzymes in gluconeogenesis, which had similar expression pattern in TAMs and M1 macrophages, the expression and activity of PCB in M1 or M_LPS_ macrophages were in stark contrast to that in TAMs (Fig. 1a, Supplementary Fig. 1b, g–k), suggesting that PCB is more likely to play a role in shaping the anti- and pro-tumor actions of macrophages. Constitutively decreased PCB levels in TAMs suggested that tumor-derived factors might induce PCB reduction, but tumor-conditioned medium (TCM) increased PCB robustly in vitro (Fig. 1d). Considering that hypoxia is a key feature of solid tumors, we exposed macrophages to 0.5% oxygen and found that combination of TCM and hypoxia suppressed PCB remarkably, while hypoxia alone did not (Fig. 1d, supplementary Fig. 1l). Consistently, the abundance of PCB in TAMs located in high-HIF1α-expressing areas was lower than in those located in low-HIF1α-expressing areas (Fig. 1e, Supplementary Fig. 1m). By fractionating TCM, we found that both < 3KDa and >3KDa fractions could suppress PCB (Supplementary Fig. 1n). Together, these data suggest that multiple factors derived from tumor contributes to the reduction of PCB in TAMs under hypoxic conditions.

**Fig. 1:**
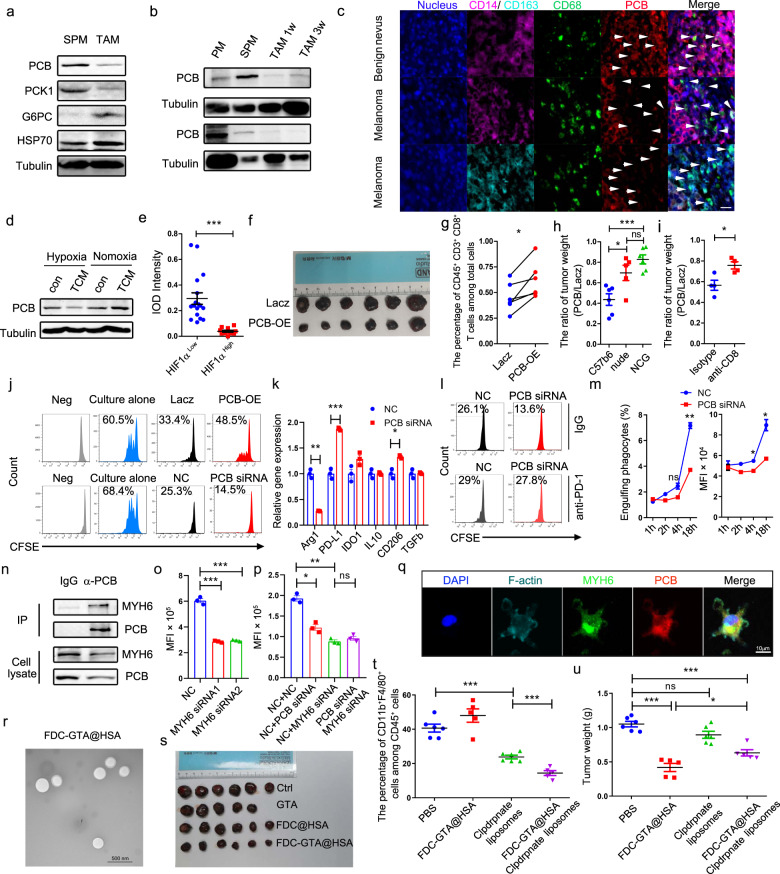
Intervening pyruvate carboxylase stunts tumor growth by strengthening anti-tumor actions of tumor-associated macrophages. **a** Analysis the protein expression of gluconeogenic enzymes PCB, PCK1 and G6PC in SPMs and TAMs. **b** Immunoblotting of PCB in peritoneal macrophages (PM), SPMs, and TAMs isolated from tumors that grow for 1 week and 3 weeks. **c** Representative immunofluorescent staining of nucleus, CD14, CD163, CD68, and PCB in the human melanoma TMA. Normal tissue n=14, melanoma n=69. Scale bar, 20 μm. **d** Immunoblotting of PCB in macrophages treated with or without TCM under normoxic or hypoxic condition. **e** Quantification of PCB expression in TAMs located in HIF1α high-expressing area or HIF1α low-expressing area in tumor sections. **f** Lacz or PCB-OE iBMDMs were inoculated with B16 cells into the skin of the left and right hind legs of the same C57BL/6 mouse and 19 days later the size of tumors was assessed. **g** The percentage of CD45^+^ CD3^+^ CD8^+^ T cells among total cells in tumor digests from indicated groups. **h** The ratio of tumor weight between PCB-OE group and Lacz group in C57BL/6 mice, nude mice, and NSG mice. **i** The ratio of tumor weight between PCB-OE group and Lacz group treated with isotype IgG or anti-CD8 antibody. **j** The proliferation of CD8^+^ T cells cultured alone or cocultured with indicated TAMs. **k** The immunosuppressive genes of TAMs transfected with scramble or PCB siRNA were analyzed RT-qPCR. **l** T cells were co-cultured with TAMs transfected with scramble or PCB siRNA in the presence or absence of PD-1-neutralizing antibody. **m** Phagocytosis of B16-EGFP by TAMs was analyzed by Flow cytometry. **n** Cell extracts from macrophages were immunoprecipitated with anti-PCB antibody, then analyzed with anti-MYH6 and anti-PCB antibodies. **o** Phagocytosis of TAMs transfected with control or MYH6 siRNA were quantified by flow cytometry. **p** Phagocytosis of TAMs transfected with indicated siRNAs. **q** Co-localization of PCB, MYH6, and F-actin in TAMs was evaluated by confocal analysis. Scale bar, 10 μm. **r** SEM images of FDC-GTA@HSA. Scale bar, 500 nm. **s** The size of tumors from mice treated with PBS, GTA, FDC@HSA, or FDC-GTA@HSA. **t**, **u** The frequency of macrophages among CD45^+^ cells (**t**) and tumor weight (**u**) of indicated groups. Data represents as mean ± SEM. *P* < 0.05, *; P < 0.01, **; *P* < 0.001, ***. Significance was calculated with student’s *t* test and paired *t* test (**g**). Experiments were repeated at least twice to observe concordant statistical significance

Next, we investigated whether intervening PCB in TAMs could influence tumor progression and anti-tumor immunity. As PCB suppression could be reversed by alleviating hypoxia (Fig. 1d), we treated tumor-bearing mice with oxygen-delivering nanoparticle FDC@HSA.^4^ As expected, FDC@HSA relieved tumor hypoxia and upregulated PCB (Supplementary Fig. 2a, b). Importantly, FDC@HSA delayed tumor growth and elevated the infiltration of CD45^+^ immune cells, cytotoxic T cells, and NK cells (Supplementary Fig. 2c–g), indicating strengthening of anti-tumor immunity. To further confirm the role of PCB in enhancing the anti-tumor actions of TAMs directly, we constructed Lacz- and PCB-overexpressing immortal bone marrow-derived macrophages (iBMDMs) and inoculated them into the same mouse along with melanoma cells (Supplementary Fig. 3a, b). PCB-OE iBMDMs stunted tumor growth significantly compared with Lacz iBMDMs (Fig. 1f, Supplementary Fig. 3c, d). To determine whether PCB-OE iBMDMs reprogramed the tumor microenvironment, we analyzed the infiltration of immune cells (Supplementary Fig. 3e, f). More CD8^+^ T cells infiltrated into tumors in the PCB-OE group (Fig. 1g, Supplementary Fig. 4a), while the percentage of CD45^+^ immune cells, CD3^+^ T cells, NKs and TAMs did not change evidently (Supplementary Fig. 4b–f). In contrast to CD8^+^ T cells, the infiltration of Tregs was lower in the PCB-OE group (Supplementary Fig. 4g), indicating relief of immune suppression. To determine the importance of T cells on PCB-OE iBMDM-induced tumor suppression, we co-inoculated iBMDMs with B16 cells into nude mice and NSG mice, the results showed that loss of T cells, but not other immune cells, significantly impaired the anti-tumor actions of PCB-OE iBMDMs (Fig. 1h, Supplementary Fig. 4h–i). To further confirm the role of CD8^+^ T cells, we depleted them and found that the tumor-inhibitory effect of PCB-OE iBMMDs was also significantly dampened (Fig. 1i, Supplementary Fig. 4j–l). Interestingly, PCB-OE iBMDMs delayed tumor growth in NSG mice (Supplementary Fig. 4i), indicating that iBMDMs could kill tumor cells by themselves. Together, our data suggest that PCB upregulation in macrophages promotes anti-tumor immunity, which is mainly mediated by CD8^+^ T cells and macrophages.

To determine whether PCB features the ability of TAMs to suppress or recruit CD8^+^ T cells, we performed T-cell proliferation and recruitment assay. Intervening PCB expression in TAMs significantly affected the ability of TAMs to inhibit T-cell proliferation and activation (Fig. 1j, Supplementary Fig. 5a–d), but had little effect on T cells recruitment (Supplementary Fig. 5e). To identify the factors that mediated the suppression of TAMs on T cells, we firstly detected the main T-cell suppressive molecules expressed by TAMs.^5^ Among these molecules, Arg1 and PD-L1 were the two genes most affected by PCB, but only PD-L1was regulated by PCB at the protein level (Fig. 1k, Supplementary Fig. 5f–h). The expression of PD-L1 was also increased in M2 macrophages after PCB knockdown (Supplementary Fig. 5i). Moreover, PCB and PD-L1 were negatively correlated in TAMs in human melanoma samples (Supplementary Fig. 5j). Consistently, IRF1, the transcriptional factor of PD-L1, increased significantly after PCB knockdown (Supplementary Fig. 5k). PD-1neutralisation almost completely reversed the enhanced suppressive effect brought by PCB knockdown (Fig. 1l), suggesting that PD-L1 mediated the regulation of PCB on the suppressive activity of TAMs. To determine whether PCB enzymatic activity could affect T-cell suppressive activity and PD-L1 expression of TAMs, we mutated PCB or treated TAMs with PCB allosteric activator acetyl-CoA, and found that both treatments could alter PD-L1 expression and the capacity of TAMs to suppress T cell (Supplementary Fig. 5l–q). These data suggest that PCB reduces the immunosuppressive capacity of TAMs by downregulating PD-L1, which is dependent on the activity of PCB.

PCB-OE iBMDMs could delay tumor growth in NSG mice indicated that PCB may regulate the tumor-killing activity of macrophages. We found that coculture with PCB-OE iBMDMs did not inhibit the proliferation of tumor cells (Supplementary Fig. 6a), which was consistent with the fact that PCB could not regulate the polarization and pro/anti-inflammatory factors secretion of TAMs (Supplementary Fig. 6b–e). However, PCB downregulation significantly impair the ability of TAMs to engulf tumor cells and latex beads (Fig. 1m, Supplementary Fig. 6f–k), while PCB upregulation promoted phagocytosis in vitro and in vivo (Supplementary Fig. 6l, m). Unexpectedly, PCB regulated phagocytosis independent of its’ activity (Supplementary Fig. 6n–q). Interestingly, we identified that MYH6/7 and MYH2 interacted with PCB (Supplementary Fig. 7a, Table 1). It is worth noting that MYH6/7 and MYH2 are myosin heavy chains belonging to the myosin II family, which includes MYH9, a protein has been proven to regulate phagocytosis. Further studies confirmed that PCB interacted with MYH6, but not with MYH7 or MYH2 (Fig. 1n, Supplementary Fig. 7b–f). Besides, PCB, a mitochondrial protein, could distribute in the cytoplasm, which was in accordance with its ability to interact with the cytoplasmic protein MYH6 (Supplementary Fig. 7g, h). MYH6 knockdown impaired phagocytosis and PCB knockdown could not further impair the phagocytosis of MYH6-knockdown TAMs, suggesting that MYH6 mediated the regulation of PCB on phagocytosis (Fig. 1o, p, Supplementary Fig. 7i). Co-IP and immunofluorescence staining demonstrated that MYH6 interacted with F-actin (Supplementary Fig. 7j, k). Moreover, PCB localized with MYH6 and F-actin, and promoted the interaction between MYH6 and F-actin (Fig. 1q, Supplementary Fig. 7l). These findings suggest a model in which the interaction between PCB and MYH6 promotes MYH6 binding with F-actin, which in turn strengthens phagocytosis.

Finally, we fabricated a nanoparticle FDC-GTA@HSA, which was loaded with triacetin that activates PCB (Supplementary Figs. 8, 9a). FDC-GTA@HSA possessed favorable grain size, stability, and ability to load/release oxygen (Fig. 1r, Supplementary Fig. 9b–f). Moreover, this nanoparticle possessed favorable tumor-targeting property (Supplementary Fig. 9g, h). Administration of FDC-GTA@HSA relieved tumor hypoxia and inhibited tumor growth more potently than FDC@HSA, which was in line with the higher PCB activity observed in TAMs after FDC-GTA@HSA treatment (Fig. 1s, Supplementary Fig. 10a–e). The infiltration of CD45^+^ cells, CD3^+^ T cells, cytotoxic T cells, and macrophages was significantly higher in the FDC-GTA@HSA group than in the FDC@HSA group (Supplementary Fig. 10f–j), indicating that FDC-GTA@HSA was more efficient in reprogramming immune microenvironment. By depleting TAMs (Fig. 1t), the anti-tumor activity of FDC-GTA@HSA was partially reversed, indicating that the tumor-inhibitory effect of FDC-GTA@HSA partly depends on TAMs (Fig. 1u, Supplementary Fig. 10k, l).

In conclusion, our data suggest that tumor exploits hypoxia to suppress PCB in TAMs, which in turn impedes the anti-tumor actions of TAMs and imposes immunosuppressive properties on TAMs. This study provides critical proof-of-concept evidence that restoring PCB in TAMs can be an adjunct form of immunotherapy.

## Supplementary information

Supplementary information
