# Supplementary material for: Intervening pyruvate carboxylase stunts tumor growth by strengthening anti-tumor actions of tumor-associated macrophages
Source: Signal Transduct Target Ther. 2022 Feb 2;7:34. doi: 10.1038/s41392-021-00807-w (PMC8807619; doi:10.1038/s41392-021-00807-w)
Supplement: Supplementary file 1 — Supplementary information [file 41392_2021_807_MOESM1_ESM.docx]

Supplementary Materials for

Intervening pyruvate carboxylase stunts tumor growth by strengthening anti-tumor actions of tumor associated macrophages

Yuxin Shu^1#^, Nanfei Yang^2#^, Nan Cheng^2#^, Zhengyun Zou^2^, Wenlong Zhang^2^, Yuncheng Bei^2^, Qian Shi^3^, Menghao Qin^2^, Wei-Guo Zhu^1*^, Pingping Shen^1, 2 *^.

Corresponding to: ppshen@nju.edu.cn (P. Shen), zhuweiguo@szu.edu.cn (WG. Zhu)

^1^ Guangdong Key Laboratory of Genome Instability and Human Disease, Shenzhen University International Cancer Center, Department of Biochemistry and Molecular Biology, Shenzhen University School of Medicine, Shenzhen 518060, PR China.

^2^ State Key Laboratory of Pharmaceutical Biotechnology and The Comprehensive Cancer Center, Nanjing Drum Tower Hospital, The Aﬃliated Hospital of Nanjing University Medical School, Nanjing University, Nanjing 210023, PR China.

^3^ Department of Cellular and Integrative Physiology, The University of Texas Health Science Center at San Antonio, 7703 Floyd Curl Drive, San Antonio, Texas 78229-3904, USA.

**This PDF file includes:**

Materials and Methods

Supplementary Text

Figures. S1 to S10

Tables S1

Materials

FBS, RPMI1640 and DMEM were purchased from Gibco (Carlsbad, CA). LPS, aspartate, acetyl-CoA, sodium oxamate, latex-beads, perfluorodecalin, glycerin triacetate, human serum albumin and IR775 were purchased from Sigma (Santa clara, CA). MCSF and IFNg were purchased from Genescript (Nanjing, China). Clodronate liposomes were purchased from Liposoma (Amsterdam, Netherlands). CFDA-SE was purchased from Beyotime (Shanghai, China). GSK2837808A was purchased from MCE (Shanghai, China). Anti-mouse/human PCB (16588-1-AP), anti-PCK1(16754-1-AP), anti-MYH2 (66212-1-Ig) and anti-MYH6 (22281-1-AP) antibodies were purchased from Proteintech (Rosemont, USA). Anti-HSP70 antibody (BS9807M) was purchased from Bioworld (Bloomington, USA). Anti-a-tubulin antibody (A01410) was purchased from Genescript (Nanjing, China). Anti-HA (sc-7392) and anti-FLAG (sc-166355) antibodies were purchased from Santa Cruz Biotechnology (Dallas, TX, USA). HRP conjugated goat anti-mouse/rabbit antibodies were purchased from Beyotime (Shanghai, China). Anti-CD45-FITC (553079), anti-CD3-PE (553063), anti-CD4-FITC (557307) were purchased from BD Bioscience (San Jose, CA). Anti-CD8-APC (17-0081-82), anti-F4/80-APC (17-4801-82), anti-CD11b-PE (12-0112-81), anti-NK1.1-APC (17-5941-82), anti-CD86-FITC (11-0862-82) were purchased from ebioscience (San Diego, CA). Anti-CD206-PE (141705) was purchased from Biolegend (San Diego, CA). Anti-mouse CD8α-InVivo was purchased from Selleck.

**Methods**

Cell culture

B16 and HEK293T were purchased from the Cell Bank of Typical Culture Preservation Committee of the Chinese Academy of Science. iBMDM was a kind gift from professor Feng Shao (National Institute of Biological Sciences, Beijing, China). The cells were cultured in DMEM or RPMI1640 with 10% FBS and with 1% penicillin-streptomycin. Bone marrow‐derived macrophages (BMDM) were obtained from mice following a specific isolating protocol. Briefly, femurs from 8‐week‐old mice were cut in a sterile glass and the bone marrow cavity was flushed out with chilled PBS using a 10‐ml syringe with 25‐G needle. Once the cells were isolated and centrifuged at 500 g, cells were cultured in DMEM containing 10% fetal bovine serum, and 1% penicillin/streptomycin. In addition, to initiate macrophage differentiation, the medium was supplemented with 100 ng/ml of M‐CSF for 7 days. During differentiation, the medium was replaced every 2 days to eliminate contaminating non‐adherent cells.

Mice

6-8 weeks old male C57bl/6 mice (19-20 g), nude mice and M-NCG mice were purchased from the Model Animal Research Center of the Nanjing University, Nanjing, China and bred in our animal facilities under specific pathogen‐free conditions. All animal experiments were approved by the Institutional Animal Care and Use Committee, Nanjing University (IACUC-1909004). Vendor health reports indicated that the mice were free of known viral, bacterial, and parasitic pathogens. The animals were housed in an SPF facility with five to six mice per cage and sterile wood shavings as bedding (12 h light/12 h dark). All the mice had free access to food and water. Animal welfare was assessed daily by the authors. All animals were either treated (where possible) or humanely euthanized at any sign of illness or stress.

Human samples

All primary melanoma tissue samples are obtained from National Human Genetic Resources Sharing Service Platform (2005DKA21300). All related procedures were performed with the approval of the institutional review and ethics boards at Nanjing Drum Tower Hospital of Nanjing University, Nanjing. No commercial sponsor was involved in this study.

Tumor models

When confluence reached 80%, cultured B16 cells and iBMDMs were harvested from the monolayer cultures, washed with serum-free medium, and re-suspended in RPMI 1640 (per ml, B16 cells :5× 10^6^ cells, iBMDMs :1× 10^6^cells), and then 20 ml cell suspension was injected into the skin of male C57bl/6 mice, nude mice or NSG mice. The animals were humanely euthanized at 19, 18 or 14 days respectively, and tumor tissues were harvested and weighed.

20 μl 4T1 cell suspension (10^7^ cells per ml) was injected into the inguinal mammary fat pads of BALB/c mice. After 30 days, the animals were humanely euthanized and the tumors were harvested for isolating TAMs.

20 μl LLC suspension (10^7^ cells per ml) was injected subcutaneously into the C57bl/6 mice. After 30 days, the animals were sacrificed and the tumors were harvested for isolating TAMs.

20 μl B16 cell suspension (5× 10^6^ cells per ml) was injected into the skin of male C57bl/6 mice. After 7 days, 20 mice are randomly assigned. Anti-CD8 antibody or Isotype IgG (Selleck, 5mg/kg) was intravenously (i.v.) administered every 3 days. Treatment was initiated when tumors became palpable on day 7 ending on day 18 post tumor implantation.

20 μl B16 cell suspension (5× 10^6^ cells per ml) was injected into the skin of male C57bl/6 mice. After 7 days, 24 mice are randomly assigned. Treatments were given as single agents with the following regimen for each drug. PBS (10 ml/kg), GTA (100 mg/kg), FDC@HSA (10 ml/kg) and FDC-GTA@HSA (10 ml/kg) was intravenously injected twice a week. Treatment was initiated when tumors became palpable on day 7 ending on day 18 post tumor implantation. Tumors were measured every four days with the vernier caliper, and the volume (0.5 ×length× width × width) was calculated.

Treatments were given as single agents or in combinations with the following regimen for each drug. PBS (10ml/kg), FDC@HSA (10ml/kg), Chlorophosphate liposomes (5ml/kg) were intravenously or intraperitoneal (Chlorophosphate liposomes) twice a week. Treatment was initiated when tumors became palpable on day 7 ending on day 17 post tumor implantation. Tumors were measured every second day with the vernier caliper, and the volume (0.5 × length × width × width) was calculated.

SPMs and TAMs isolation

Tumor tissues were digested by collagenase I and IV minced in the DMEM at 37℃ for1 hour and filtered using a 40-μm cell strainer (BD Falcon). Spleen tissue was soaked in PBS and cut into small pieces with scissors and filtered using a 40-μm cell strainer. Cells in suspensions were stained for 15 min at 4 ℃ with anti-mouse F4/80 microbeads (Miltenyi Biotec). After rigorous washing with PBS containing 0.5% BSA and 2 mM EDTA, cells were sorted by MACS separation according to the manufacturer’s instruction.

Quantitative real-time PCR

Total RNA was extracted by use of TRIzol (Invitrogen). Reverse transcription was performed with 5 × All-In-One RT Master Mix (abm) kit and quantitative PCR reactions were performed using SYBR Green Master Mix (Vazyme biotech) kit. PCRs were performed in a total volume of 20 μl. Data were collected and analyzed by CFX96 Real-Time System (Bio-Rad C1000 Touch Thermal Cycler or AB StepOnePlus™ Real-Time PCR System). The details of primers are listed as follows:

| **Gene** | **Sequences** | |
| --- | --- | --- |
| PCB | Forward | CTGAAGTTCCAAACAGTTCGAGG |
|  | Reverse | CGCACGAAACACTCGGATG |
| PD-L1 | Forward | GCTCCAAAGGACTTGTACGTG |
|  | Reverse | TGATCTGAAGGGCAGCATTTC |
| PCK1 | Forward | CTGCATAACGGTCTGGACTTC |
|  | Reverse | CAGCAACTGCCCGTACTCC |
| G6PC | Forward | CGACTCGCTATCTCCAAGTGA |
|  | Reverse | GTTGAACCAGTCTCCGACCA |
| Arg1 | Forward | CTCCAAGCCAAAGTCCTTAGAG |
|  | Reverse | AGGAGCTGTCATTAGGGACATC |
| IDO1 | Forward | GCTTTGCTCTACCACATCCAC |
|  | Reverse | CAGGCGCTGTAACCTGTGT |
| CD206 | Forward | CTCTGTTCAGCTATTGGACGC |
|  | Reverse | CGGAATTTCTGGGATTCAGCTTC |
| IL-10 | Forward | GCTCTTACTGACTGGCATGAG |
|  | Reverse | CGCAGCTCTAGGAGCATGTG |
| TGFβ | Forward | CTCCCGTGGCTTCTAGTGC |
|  | Reverse | GCCTTAGTTTGGACAGGATCTG |
| CCL5 | Forward | GCTGCTTTGCCTACCTCTCC |
|  | Reverse | TCGAGTGACAAACACGACTGC |
| CXCL9 | Forward | TCCTTTTGGGCATCATCTTCC |
|  | Reverse | TTTGTAGTGGATCGTGCCTCG |
| CXCL10 | Forward | CCAAGTGCTGCCGTCATTTTC |
|  | Reverse | GGCTCGCAGGGATGATTTCAA |
| CXCL11 | Forward | GGCTTCCTTATGTTCAAACAGGG |
|  | Reverse | GCCGTTACTCGGGTAAATTACA |
| CX3CL1 | Forward | ACGAAATGCGAAATCATGTGC |
|  | Reverse | CTGTGTCGTCTCCAGGACAA |
| RPL13A | Forward | AGCCTACCAGAAAGTTTGCTTAC |
|  | Reverse | GCTTCTTCTTCCGATAGTGCATC |

Activities of PCB and PCK1

The activities of PCB and PCK1 were determined by using Pyruvate Carboxylase activity testing kit (Solarbio) and Phosphoenolpyruvate carboxykinase 1 activity testing kit (Solarbio) respectively.

Immunofluorescence and IOD measurement

The paraffin section was deparaffinized by xylene and rehydrated by a series of ethanol solutions. Then, the section was pressure cooked for 5 min in Sodium Citrate buffer for antigen retrieval and incubated with PBS containing 0.3% Tween-100 and 5% goat serum for at room temperature 1 hour. After being blocked, the sections were incubated with a primary antibody overnight at 4 ℃. The sections were then briefly rinsed with PBS, washed 3 times, 5 min every time, and incubated with fluorescence-coupled secondary antibody (Beyotime or Bioss) for 4 h at room temperature. Nuclei staining was performed by incubating the cells with DAPI (Beyotime). The specimens were then observed at the appropriate fluorescence wavelength using a confocal microscope (Nikon A1-HD25 or Zeiss LSM 880). IOD means Integral Optical Density or Immunofluorescence Accumulation Optical Density, which is measured by Image-pro plus 6.0 (Media Cybernetics, Inc., Rockville, MD, USA). To quantify the IOD of PCB in TAMs in immunofluorescence images, images (red or green) were converted to grayscale, and the areas corresponding to the staining of F4/80, CD14, CD206 in the grayscale images were set to be AOI (area of interest), and the IOD and area sum of AOI were counted. IOD intensity = IOD/area sum.

Image acquisition and quantitative analysis (TissueGnostics)

The images were acquired by TissueFAXS (TissueGnostics GmbH, Vienna Austria) with a Zeiss Axio Imager Z2 Microscope System at ×20 （or ×40）magnification. The cell density, nucleus area per cell, area per cell and expression per cell were quantified using StrataQuest software (version 7.0.1, TissueGnostics GmbH, Vienna, Austria)

Flow cytometry analysis

Tumors (about 0.5g) were sniped and digested. All cells collected after tumors were digested were pre-incubated with FcR blocking antibody (Miltenyi Biotec) for 15 min at 4℃ at a concentration of 1 mg/ 1×10^6^ cells/ 100 μl. Fluorescent antibodies and isotype antibodies were consequently added at the indicated concentration, and cells were incubated for a further 1-2 h at 4℃. Then cells were washed twice with ice-cold PBS. The cells were analyzed on BD cytometer or NovoCyte Flow Cytometer Systems using FlowJo software (V. 7.6.4, TreeStar).

Synthesis and characterization of FDC-GTA@HSA nanoparticles

135 mg Human serum albumin was dissolved in 2.7 ml deionized water. Then, 0.3 ml Perfluorodecalin (FDC) was added to HSA solution. Finally, 100 ml GTA mixed with 200 μl ethyl alcohol was added gradually to the solution followed by gently blending for 2 min. The mixture was sonicated under 290 W for 8 min in an ice bath to form FDC-GTA@HSA. Dynamic light scattering (Brookhaven Instrum. Corp) was used to measure the size and zeta potential of FDC-GTA@HSA. The morphology and size of FDC-GTA@HSA were characterized by scanning electron microscopy.

Oxygen loading and release

1 ml of FDC-GTA@HSA nanoparticles was stored in an aseptic oxygen chamber (Oxygen flow rate= 5 L/min) for 5min to achieve oxygen saturation using a reported method.

To measure oxygen release, 1ml of oxygen saturated FDC-GTA@HSA was added into 4 ml of deoxygenated water placed in a 10 ml of centrifuge tube. Meanwhile, the oxygen concentrations were measured by a portable dissolved oxygen meter (Rex, JPB-607A) every 6 minutes.

RNA interference

The RNAi oligonucleotide sequences including non-specific siRNA: 5’-UUCUCCGAACGUGUCACGU-3’, PCB siRNA1 sense strand: 5’-GCUGAUGAAGCCUACCUUA-3’ PCB siRNA2 sense strand: 5’-CGUCUGGAGUAUAAGCCUA-3’, MYH6 siRNA1 sense strand: 5’-GAAAGACGGUGACCAUAAA-3’, MYH6 siRNA2 sense strand: 5’- GACAAGGAGGAGUAUGUUA-3’were all purchased from Gene Pharma (Shanghai). BMDMs were transfected with RNAi oligonucleotides using RNAiMAX and incubated for 24hours before treatments.

CRISPR-CAS9

gRNA designed to target the common exons for all mice PCB isoforms were synthesized as follows: oligo #1, 5′- CACCGTACTCCAGACGCCGGACATT-3′, oligo #2, 5′- CACCGTAGGCTTATACTCCAGACGC-3′, and cloned to lentiCRISPRv2 plasmids.

Co-IP

For the immunoprecipitation assays, whole-cell extracts were prepared after transfection, incubated overnight with the appropriate antibodies and subsequently incubated with Protein A/G beads (Beyotime) for 2 h. The beads were then washed 5 times with low-salt lysis buffer, and the immunoprecipitates were eluted form beads in 1 × SDS Loading Buffer (Beyotime) and boiling for 10 min, and then resolved by SDS-PAGE. Proteins were transferred to PVDF membranes (Bio-Rad) and incubated with the appropriate antibodies. The LumiGlo Chemiluminescent Substrate System (Thermo Fisher Scienfitic) was used for protein detection.

Mass spectrometry

After immunoprecipitation, the protein bands were excised in our laboratory by using trypsin-based in-gel protein digestions, and the subsequent MS analysis was conducted at the School of Life Sciences, Peking University.

T cell proliferation assay

Splenic CD8^+^ T cells were isolated by anti-CD8a particles (BD IMag) and labeled with 0.5 mM CFSE (Thermo Fisher) for 15 min at room temperature and incubated with indicated TAMs (10:1) in RPMI 1640 medium supplemented with 10 mg/ml anti-CD3 antibody, 5 mg/ml anti-CD28 antibody, and 10% FBS. Proliferation of CD8+ T cells was measured by CFSE staining and flow cytometry after 3 days. In some experiments, 5 mg/ml anti- PD-1 neutralizing antibody, 5 mg/ml IgG control was added in the co-culture.

Phagocytosis assay

Phagocytosis assays were conducted using fluorescent red latex beads (1 μM diameter, L-2778, Sigma-Aldrich) or EGFP-B16 cells. Latex beads were opsonized with complete medium (10% FBS in PRMI 1640) for 1 h at 37 °C before the phagocytosis assays. Opsonized beads were added to macrophage cells at a ratio of 10:1 and incubated at 37 °C for 0.5-2 h. Phagocytosis was terminated with the addition of 1 ml ice-cold sterile PBS. Cells were harvested and washed in ice-cold PBS 3 times andanalyzed by using flow cytometry. To measure the capacity of TAMs to engulf tumor cells, 1 × 10^5^ TAMs were plated per well in a 24-well culture plate and incubated in serum-free medium for 2 h, and then 1 × 10^6^ B16-EGFP tumor cells were added and incubated for 1-18 h. Macrophages were repeatedly washed and phagocytosis was then determined by flow cytometry detection of F4/80^+^TAMs. The phagocytic index was calculated by mean/medium fluorescence intensity (MFI) of EGFP.

Statistical Analysis

The data were expressed as the mean ± standard error of mean (SEM). The statistical analysis was performed by the paired Student's t-test, unpaired Student's t-test or one-way ANOVA followed by a Tukey's multiple comparisons. P < 0.05, P < 0.01 or P < 0.001 were considered statistically significant and indicated by *, ** or ***, respectively.

Supplementary Text

Ethics approval and consent to participate

This study was performed with the approval of the institutional review and ethics boards at Nanjing Drum Tower Hospital of Nanjing University, Nanjing. No commercial sponsor was involved in this study. All mouse procedures and experiments for this study were approved by the Institutional Animal Care and Use Committee at Nanjing University, Nanjing.

Consent for publication

Not applicable.

Availability of supporting data

The data used and analyzed in this study are available in the main text and the Supplementary Materials. Any other raw data that support the findings of this study are available from the corresponding author upon reasonable request.

Competing Interests

The authors declare no competing interests.

Funding

This work was supported by grants from the Key Research and Development Program of Jiangsu Province-Social Development Projects (BE2020687), the National Key Research and Development Program of China (2017YFA0506000), the National Natural Science Foundation of China (81673439, 82073365) and the Open Project of State Key Laboratory of Natural Medicines (SKLNMKF202109).

Author Contributions

Shen P. supervised the project and wrote the manuscript; Zhu WG. supervised the project and modified the manuscript. Shu Y., Yang N. and Cheng N. designed the study, performed most experiments and data analysis; Z Zou provided human melanoma samples; Zhang W., Bei Y., and Qing M. performed experiments; Qin Shi modified the manuscript. All authors critically reviewed the article and approved the final manuscript.

Acknowledgment

We thank Prof. Feng Shao (National Institute of Biological Sciences, Beijing, 102206,

China) for kindly providing iBMDM cell line. We thank members of Shen’s laboratory for helpful discussions, technical assistance and critical reading of the manuscript. We are grateful to TissueGnostics Asia Pacific Ltd for their technical support. We would like to give our special thanks to Mr. Hai Wu, Mr. Guangchao Li and Mr. Yang Li for their support in TissueGnostics analysis.

Figure. S1.


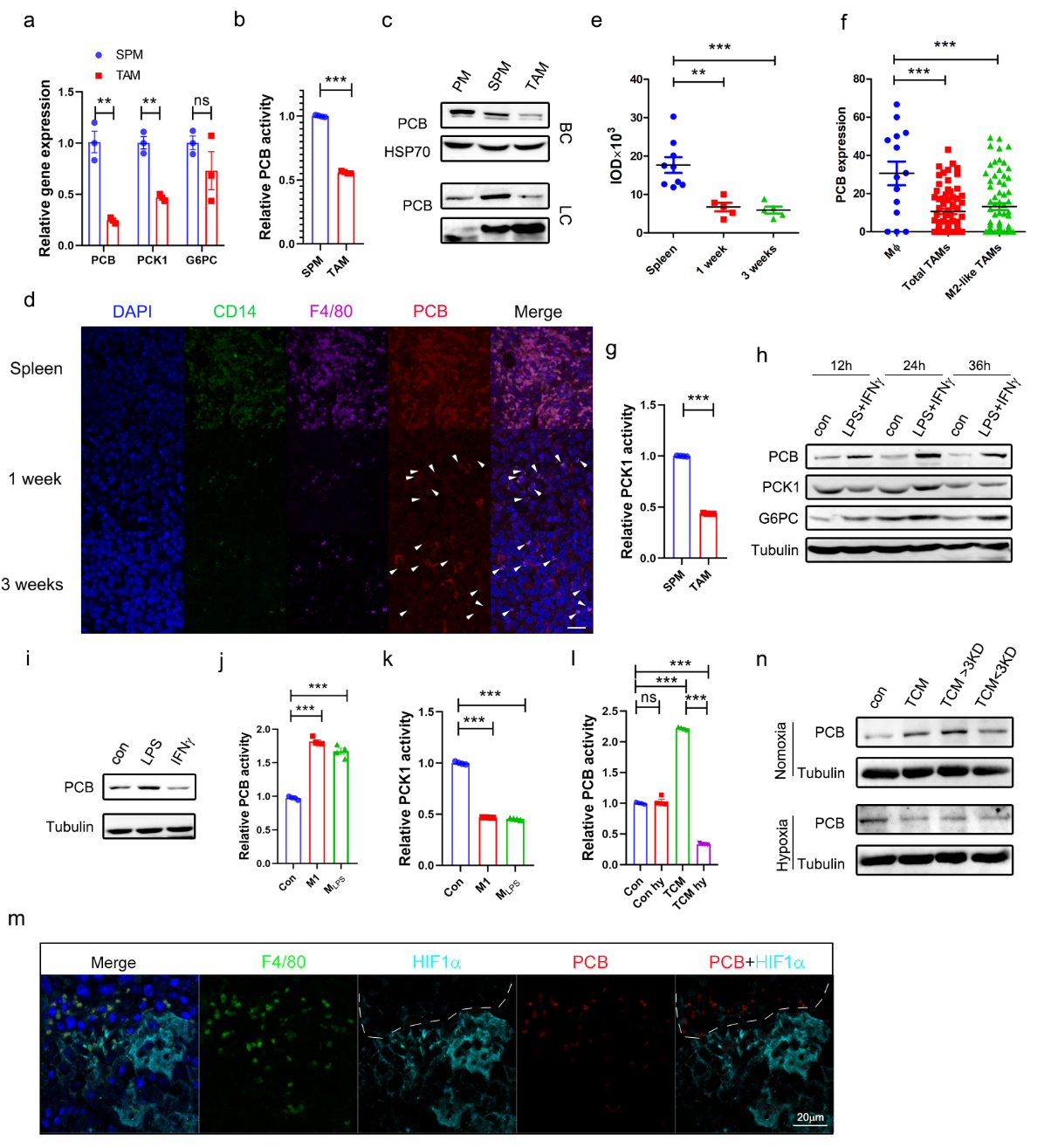


**Fig. S1** PCB in TAMs is suppressed. **(a)** Analysis the mRNA expression of gluconeogenic enzymes PCB, PCK1 and G6PC in SPMs and TAMs. **(b)** Relative PCB activities in SPMs and TAMs. **(c)** Immunoblotting of PCB in peritoneal macrophages (PMs), SPMs and TAMs isolated from 4T1 tumor-bearing (BC) or LLC tumor-bearing mice (LC). **(d)** Representative immunofluorescent staining of DAPI, PCB, F4/80 and CD14 in the sections of resected spleens and tumors (grow for 1week and 3weeks) from melanoma-bearing mice. Scale bar, 20 μm. The white tips indicate macrophages **(e)** Quantitation of (d). Spleen n=9, tumor (1week) n=5, tumor (3 weeks) n=5. IOD: Integral Optical Density **(f)** Quantitation of PCB expression in macrophages in melanoma TMA by TissueGnostics. normal tissue n=14, melanoma n=69. **(g)** Relative PCK1 activities in SPMs and TAMs. **(h)** Immunoblotting of PCB, PCK1, G6PC in macrophages treated with vehicle or LPS (50 ng/ml) and IFNγ (20 ng/ml) for indicated times. **(i)** Immunoblotting of PCB in macrophages treated with vehicle, LPS, or IFNγ for 24h. **(j, k)** Relative PCB (j) and PCK1 (k) activities in indicated macrophages. **(l)** PCB activity in macrophages treated with or without TCM under normoxic or hypoxic condition. **(m)** Representative immunofluorescent staining of DAPI, PCB, F4/80, HIF1α in the sections of resected melanoma samples from melanoma-bearing mice. Scale bar, 20 μm. **(n)** Immunoblotting of PCB in macrophages treated with unfractionated TCM (whole), TCM<3 KDa or TCM>3 KDa fractions under normoxic or hypoxic condition. Data represent as mean ± SEM. P<0.05, *; P<0.01, **; P<0.001, *** by Student’s t test and One-way ANOVA (e) and (f). Experiment was repeated at least twice to observe concordant statistical significance.

Figure. S2.


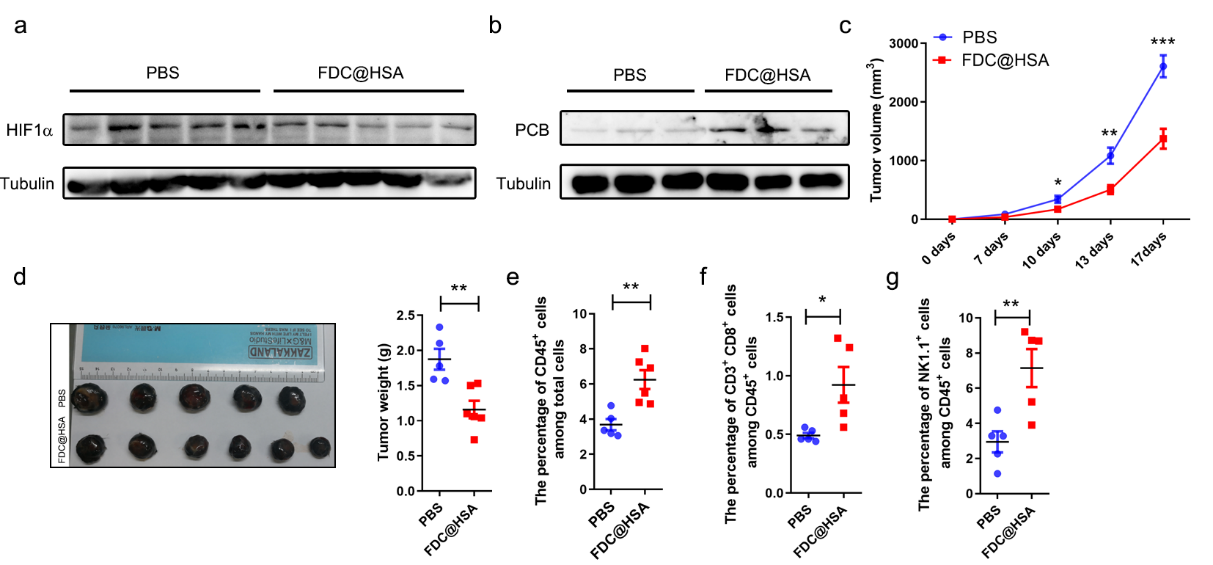


**Fig. S2** In vivo evaluation of FDC@HSA. B16 cells were inoculated into the skin of C57BL/6 mouse. After 3 days, mice were treated with PBS or FDC@HSA via tail vein injection twice a week, and 17 days later, mice were sacrificed. **(a)** Immunoblotting of HIF1α in tumor lysates from tumors of PBS or FDC@HSA treated mice. **(b)** Immunoblotting of PCB in TAMs isolated from tumors of PBS or FDC@HSA treated mice. **(c)** Growth curve of tumors from mice treated with PBS or FDC@HSA. **(d)** The size (left) and the weight (right) of tumors were assessed. **(e)** Frequency of CD45^+^ immune cells among total cells (**f, g**), CD3^+^ CD8^+^ T cells (f), NK1.1^+^ NK cells (g) among CD45^+^ cells in tumor digests from indicated groups. Data represents as mean ± SEM, P<0.05, *; P<0.01, **; P<0.001, *** by Student’s t test.

Figure. S3.


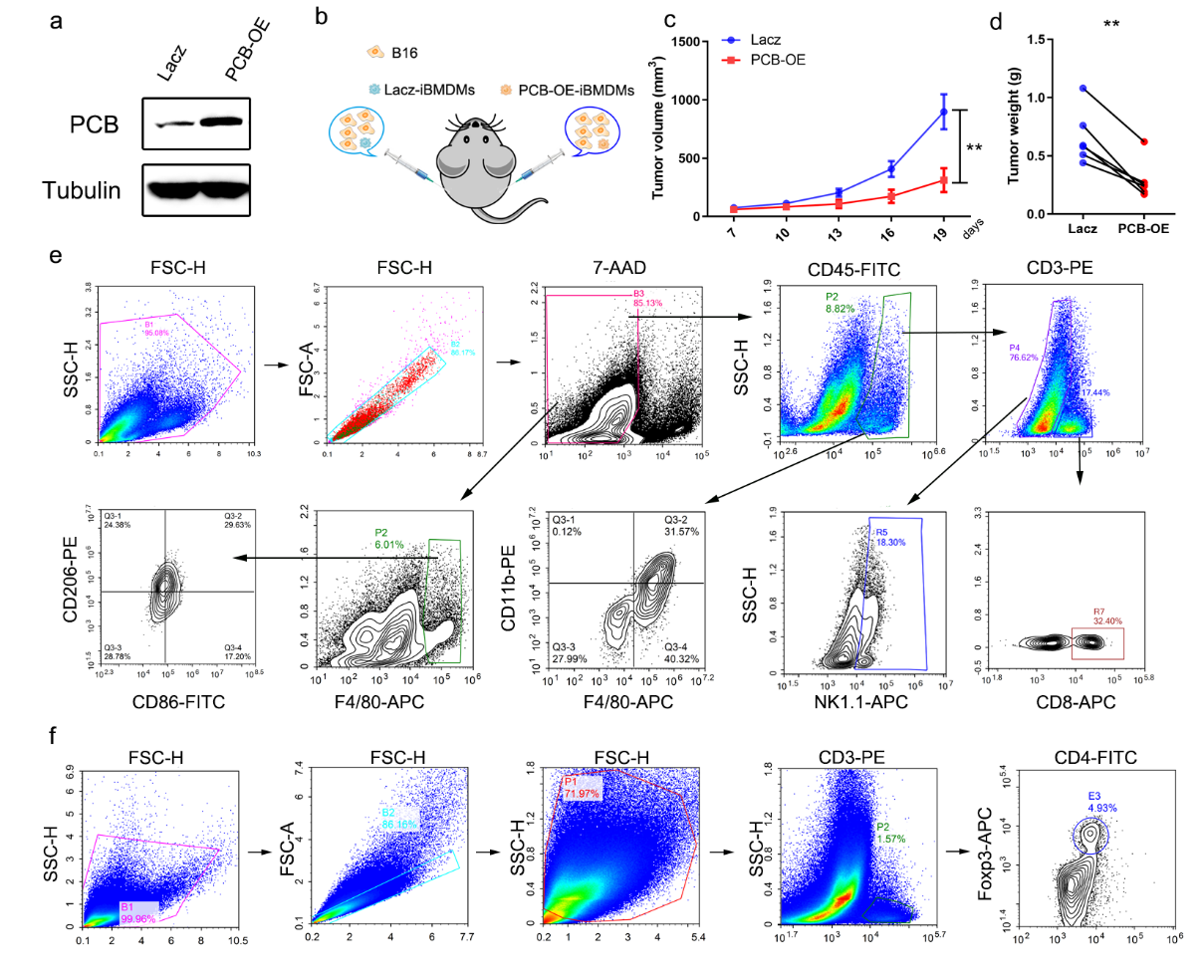


**Fig. S3** PCB-OE iBMDMs inhibit tumor growth and gating strategy of flow cytometry. **(a)** Immunoblotting of PCB in Lacz and PCB-OE iBMDMs. **(b)** Diagram showing the approach used to inoculate iBMDMs and B16 into the skin of mice. **(c, d)** Lacz or PCB-OE iBMDMs were inoculated with B16 cells into the skin of the left and right hind legs of the same C57BL/6 mouse and 19 days later, mice were sacrificed. The growth curve (c) and the weight (d) of tumors were assessed. **(e)** Gating strategy for CD8^+^ T cells, TAMs, M1 TAMs, M2 TAMs and NK cells. **(f)** Gating strategy for Tregs. Data represents as mean ± SEM, P<0.01, **. Significance was calculated with Student’s t test and paired t test (d).

Figure. S4.


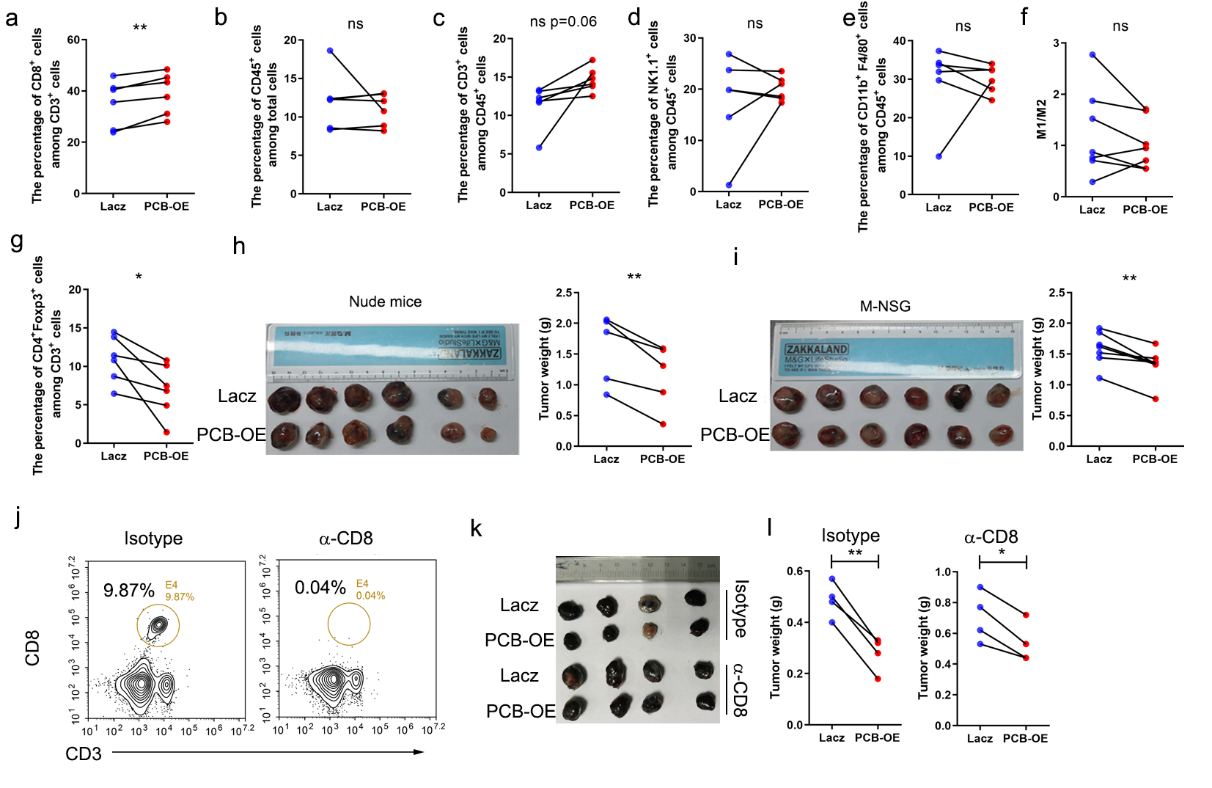


**Fig. S4** CD8^+^ T cells and iBMDMs mediate the tumor-inhibitory effect of PCB upregulation in macrophages. **(a)** Frequency of CD8^+^ cytotoxic T cells among CD3^+^ T cells in Lacz and PCB-OE tumors. **(b)** Frequency of CD45^+^ immune cells in tumors from Lacz and PCB-OE group. **(c)** Frequency of CD3^+^ T cells among CD45^+^ immune cells. **(d)** Frequency of CD3^-^ NK1.1^+^ NK cells among CD45^+^ immune cells. **(e)** The percentage of CD45^+^ F4/80^+^ CD11b^+^ macrophages among CD45^+^ immune cells. **(f)** The M1/M2 ratio of TAMs in Lacz and PCB-OE tumors. **(g)** Frequency of CD4^+^ Foxp3^+^ Tregs among CD3^+^ T cells. **(h)** Lacz or PCB-OE iBMDMs were inoculated with B16 cells into the skin of nude mice and the size (left) and the weight (right) of tumors were assessed 18 days later. **(i)** Lacz or PCB-OE iBMDMs were inoculated with B16 cells into the skin of NSG mice and the size (left) and the weight (right) of tumors were assessed 14 days later. **(j-l)** Lacz or PCB-HA overexpressing iBMDMs were co-inoculated with B16 cells into the skin of the left and right hind legs of the same mouse. After 7 days, mice were randomly assigned to receive the intravenous injection (i.v.) of isotype IgG or CD8 antibodies every 3 days. **(j)** Flow cytometry analysis of CD8^+^ T cells (Pre-gated on CD45^+^ cells) in the blood of mice. **(k, l)** Tumor size (k) and weight (j) of indicated group. Data represents as mean ± SEM, P<0.05, *; P<0.01, **; P<0.001, ***. Significance was calculated with paired t test.

Figure. S5.


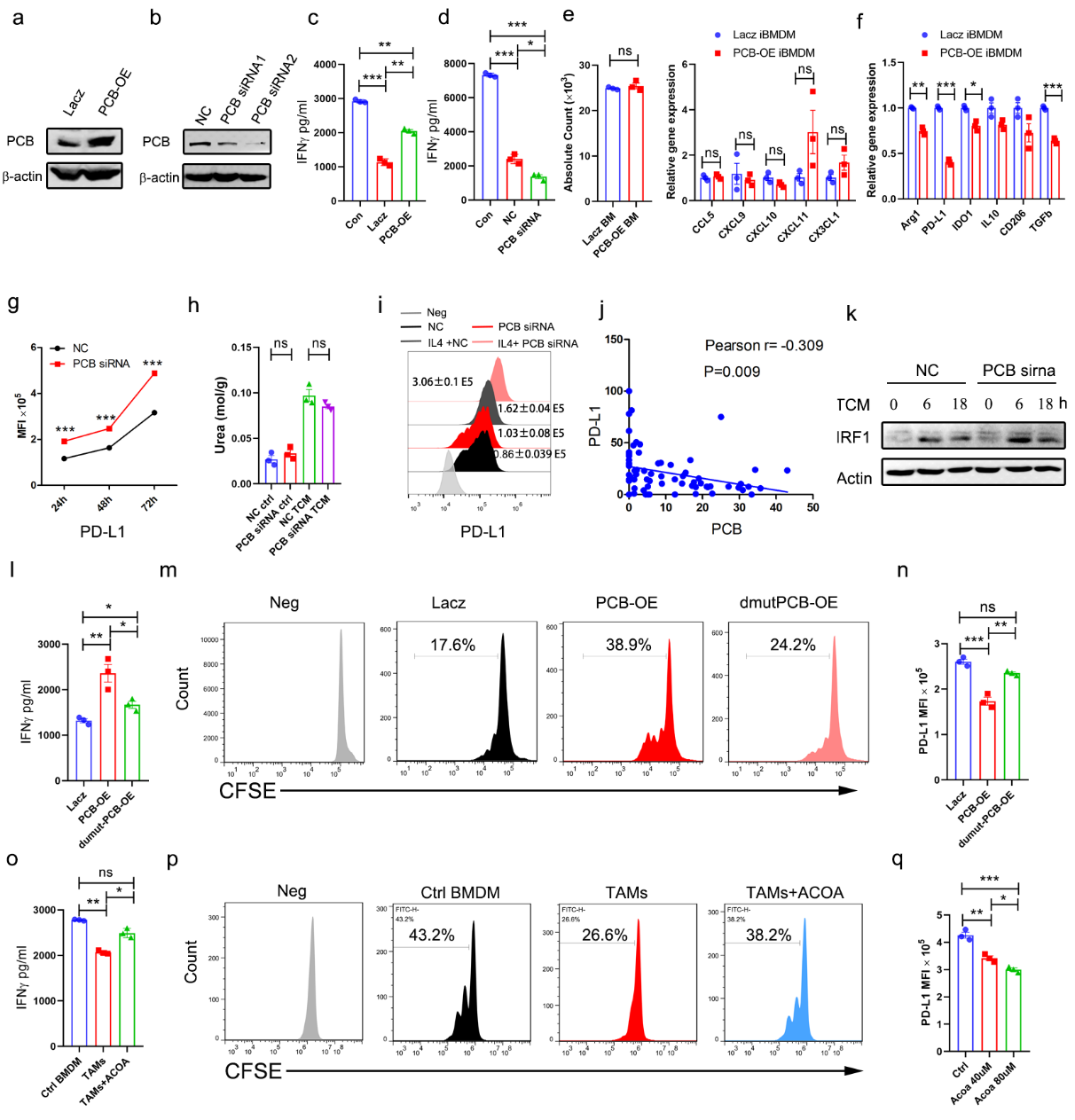


**Figure. S5** PCB regulates PD-L1 expression and immunosuppressive activity of TAMs. **(a, b)** Immunoblotting of PCB in PCB in TAMs infected by lentivirus expressing Lacz or PCB (a), and macrophages transfected with NC, PCB siRNA1 or PCB siRNA2 (b). **(c)** The IFNγ production of CD8^+^ T cells cocultured with Lacz- or PCB-overexpressing TAMs. **(d)** The IFNγ production of CD8^+^ T cells cocultured with TAMs transfected with NC or PCB siRNA. **(e)** The ability of Lacz and PCB-OE iBMDMs treated with TCM under hypoxia to recruit T cells (right) and the expression of chemokines (left) in these macrophages were analyzed. **(f)** Lacz and PCB-OE iBMDMs treated with TCM under hypoxia were assayed for indicated gene mRNA expression by RT-qPCR. **(g)** PD-L1 expression on TAMs transfected with NC or PCB siRNA was quantified by flow cytometry. **(h)** BMDMs transfected with control or PCB siRNA were cultured under normoxia in the absence or presence of TCM (15% v/v), the arginase activity was quantified by arginase activity assay kit. **(i)** BMDMs transfected with control or PCB siRNA were treated with IL4 for 24 hours, the expression of PD-L1 was analyzed. **(j)** Correlation analysis of PCB and PD-L1 protein level in human TAMs. The abundance of PCB and PD-L1 in TAMs was quantified using TissueGnostics. Melanoma specimens n=69. (**k**) Immunoblotting of IRF1 in control and PCB knockdown macrophages treated with TCM for indicated times. **(l, m)** The IFNγ production (l) and proliferation (m) of CD8^+^ T cells cocultured with TAMs overexpressing Lacz, wild-type PCB or mutant PCB. **(n)** PD-L1 expression on TAMs overexpressed Lacz, wild-type PCB or mutant PCB. **(o, p)** The IFNγ production (o) and proliferation (p) of CD8^+^ T cells cocultured with resting BMDMs, TAMs or TAMs pretreated with acetyl-CoA (80 μm). **(q)** PD-L1 expression on TAMs treated with vehicle or with different concentrations of acetyl-CoA. Data represents as mean ± SEM, P<0.05, *; P<0.01, **; P<0.001, *** by Student’s t test.

Figure. S6.


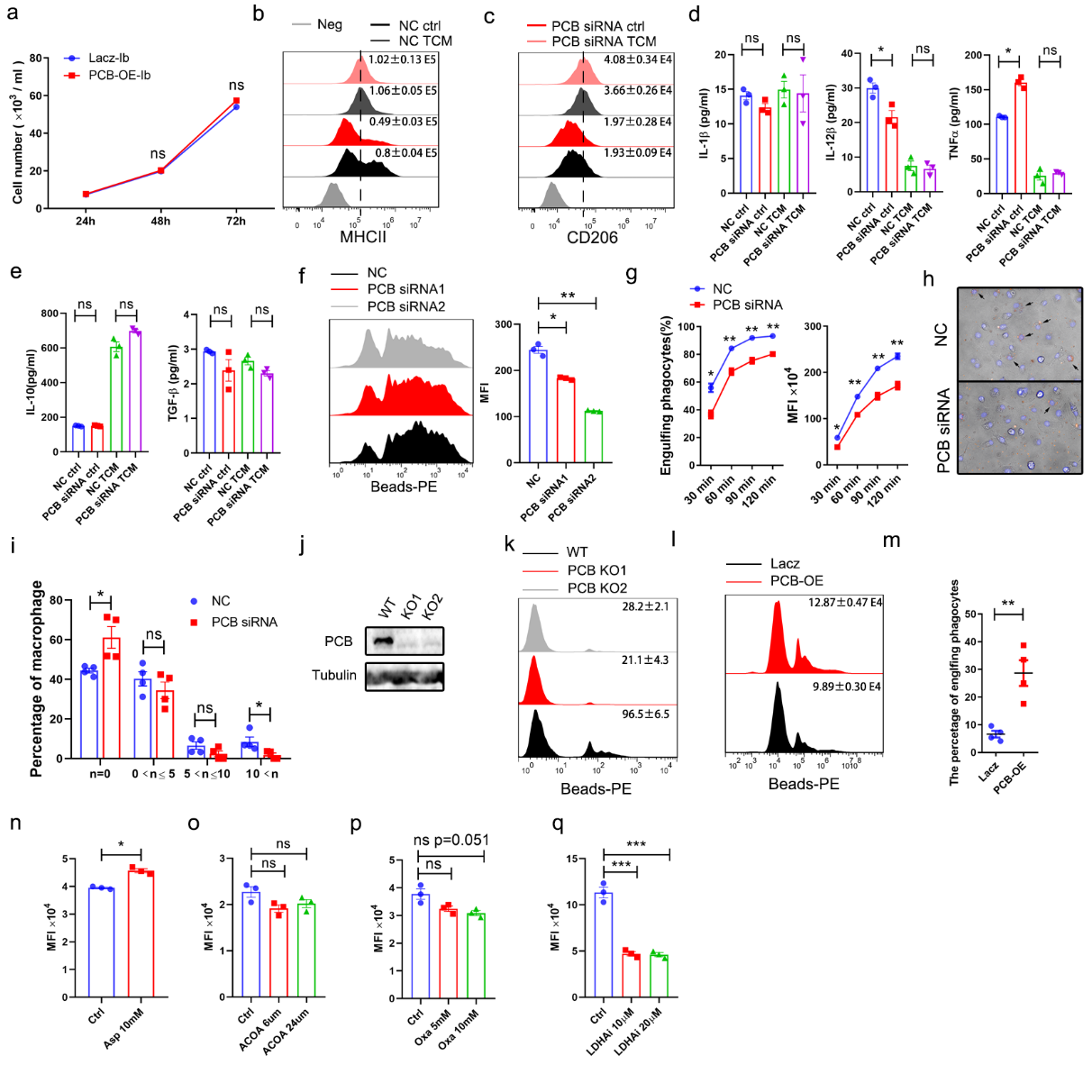


**Fig. S6** TAMs phagocytosis is regulated by PCB. **(a)** The growth curve of B16 tumor cells cocultured with Lacz iBMDMs or PCB-OE iBMDMs. **(b-e)** BMDMs transfected with control or PCB siRNA were cultured under normoxia in the absence or presence of TCM (15% v/v). **(b, c)** The pro-inflammatory maker MHCII (b) and anti-inflammatory marker CD206 (e) were analyzed by flow cytometry. **(d, e)** The expression of pro-inflammatory factors IL1β, IL12β, TNFα (d) and anti-inflammatory factors IL10, TGFβ (e) were analyzed by ELISA. **(f)** Phagocytosis of latex-beads by TAMs transfected with indicated siRNAs were analyzed by flow cytometry. **(g)** TAMs transfected with indicated siRNAs were incubated with latex-beads for 30, 60, 90, 120 minutes, and the percentage (left) and MFI of engulfing phagocytes (right) were shown. **(h)** Comparison of indicated TAMs engulfing latex-beads (1h) assessed by Live Cell Imaging System. Arrowheads indicate TAMs that engulfed more than 10 beads. **(i)** Quantification of (h). **(j)** Immunoblotting of PCB in wild type and PCB-knockout iBMDMs. **(k)** Phagocytosis of control and PCB-KO iBMDMs treated with TCM under normoxia. **(l)** Phagocytosis of Lacz and PCB-OE iBMDMs treated with TCM under hypoxia. (**m**) Lacz or PCB-HA overexpressing iBMDMs were co-inoculated with B16-GFP cells intradermally into mice. After 11 days of inoculation, the mice were sacrificed. The phagocytic capacity of Lacz or PCB-HA overexpressing iBMDMs was analyzed by FACS. **(n-r)** Phagocytosis of TAMs treated with PCB allosteric inbibitor aspirate (n), allosteric activator acetyl-CoA (o), competitive inhibitor sodium oxamate (p), or LDHA inhibitor GSK2837808A (q) for 24h. Data represents as mean ± SEM. Experiment were repeated at least twice to observe concordant statistical significance. P<0.05, *; P<0.01, **; P<0.001, *** by Student’s t test.

Figure. S7.


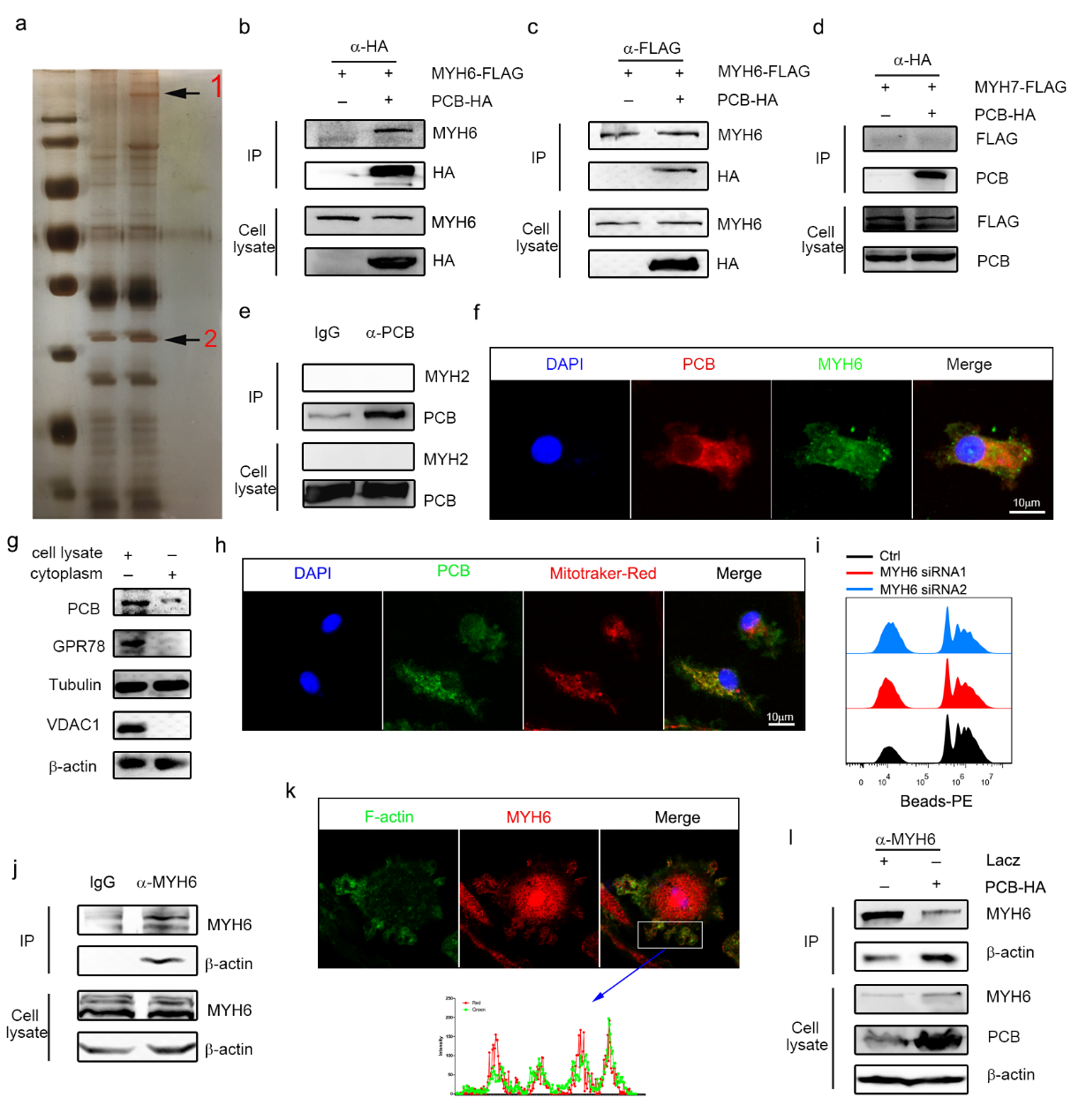


**Fig. S7** MYH6 mediates the regulation of PCB on phagocytosis. **(a)** Representative silver-stained gel showing examples of the cellular proteins that specifically interact with PCB (band 1 and 2). **(b, c)** MEFs were co-transfected with MYH6-FLAG and Lacz or PCB-HA. Cell extracts were immunoprecipitated with anti-HA antibody (b) or anti-FLAG (c) antibody, then analyzed with anti-MYH6 and anti-HA antibodies. **(d)** MEFs were co-transfected with MYH7-FLAG and Lacz or PCB-HA. Cell extracts were immunoprecipitated with anti-HA antibody, then analyzed with anti-FLAG and anti-PCB antibodies. **(e)** Cell extracts form macrophages were immunoprecipitated with IgG or anti-PCB antibody, then analyzed with anti-MYH2 and anti-PCB antibodies. **(f)** Co-localization of PCB and MYH6 in macrophages was evaluated by confocal analysis. Scale bar, 10 μm. **(g)** Whole-cell lysate and cytoplasm without mitochondria of macrophages were analyzed by immunoblot with indicated antibodies. **(h)** Co-localization of PCB and mitochondria in macrophages was evaluated by confocal analysis. Scale bar, 10 μm. **(i)** Phagocytosis of TAMs transfected with control or MYH6 siRNA were analyzed by flow cytometry. **(j)** Cell extracts from macrophages were immunoprecipitated with anti-MYH6 antibody, then analyzed with anti-MYH6 and anti-β-actin antibodies. **(k)** Representative immunofluorescent staining of F-actin, MYH6 in pseudopodia of TAMs (up), localization of MYH6 and F-actin in TAMs was analyzed by Zen software (down). **(l)** MEFs were infected with lacz- or PCB- expressing lentivirus. After 48h, cell extracts were immunoprecipitated with anti-MYH6 antibody, then analyzed by immunoblotting.

Figure. S8.


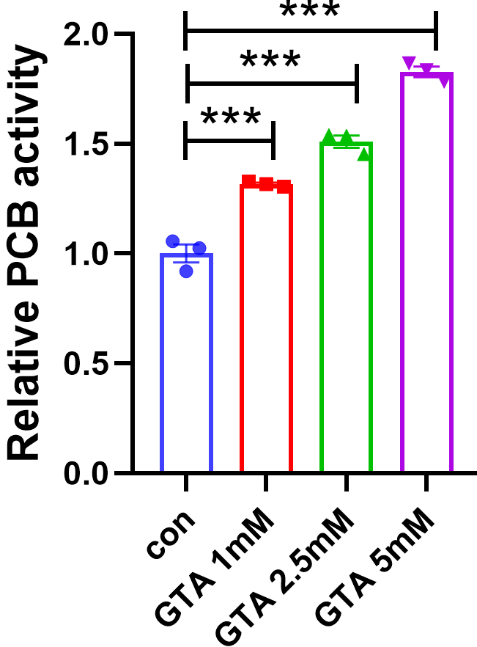


**Fig. S8** PCB is activated by GTA. Activity of PCB in BMDMs treated with indicated dose of GTA for 24 hours was measured. Data represents as mean ± SEM. Experiment were repeated at least twice to observe concordant statistical significance. P<0.001, *** by Student’s t test.

Figure. S9.


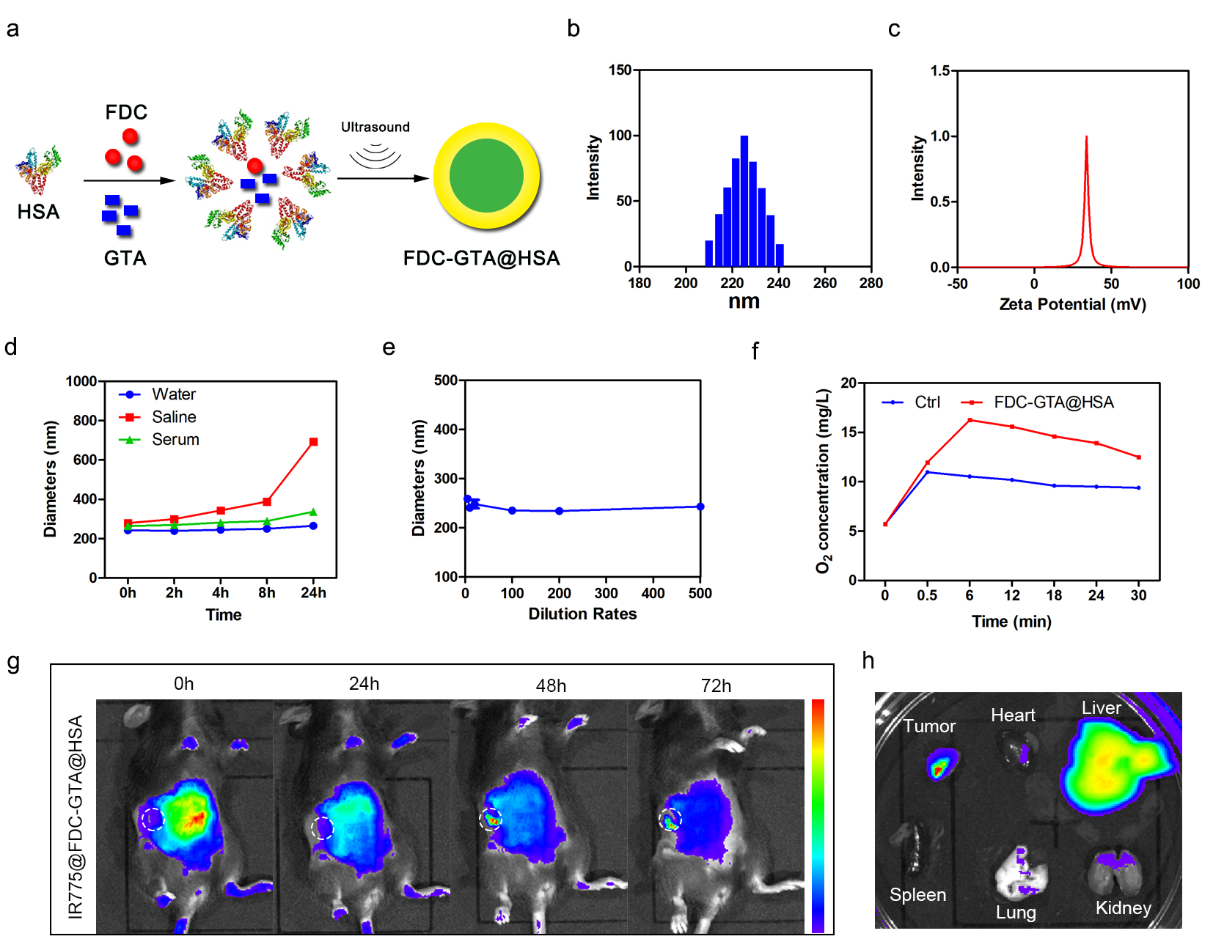


**Fig. S9** Synthesis and characterization of FDC-GTA@HSA. **(a)** A scheme showing the preparation of FDC-GTA@HSA. **(b)** FDC-GTA@HSA diameters detected via dynamic light scattering. **(c)** Zeta potential curve of FDC-GTA@HSA. **(d)** Stability of FDC-GTA@HSA diluted in different solutions: water, saline and serum. **(e)** Stability of FDC-GTA@HSA in serum after diluted for different ratios (n=3). **(f)** Oxygen concentration in deoxygenated water after addition of FDC-GTA@HSA. **(g)** In vivo fluorescence imaging of IR775 loaded FDC-GTA@HSA. **(h)** Ex vivo fluorescence image of major organs and tumors after 72 hours of FDC-GTA@HSA treatment. Data represents as mean ± SD.

Figure. S10.


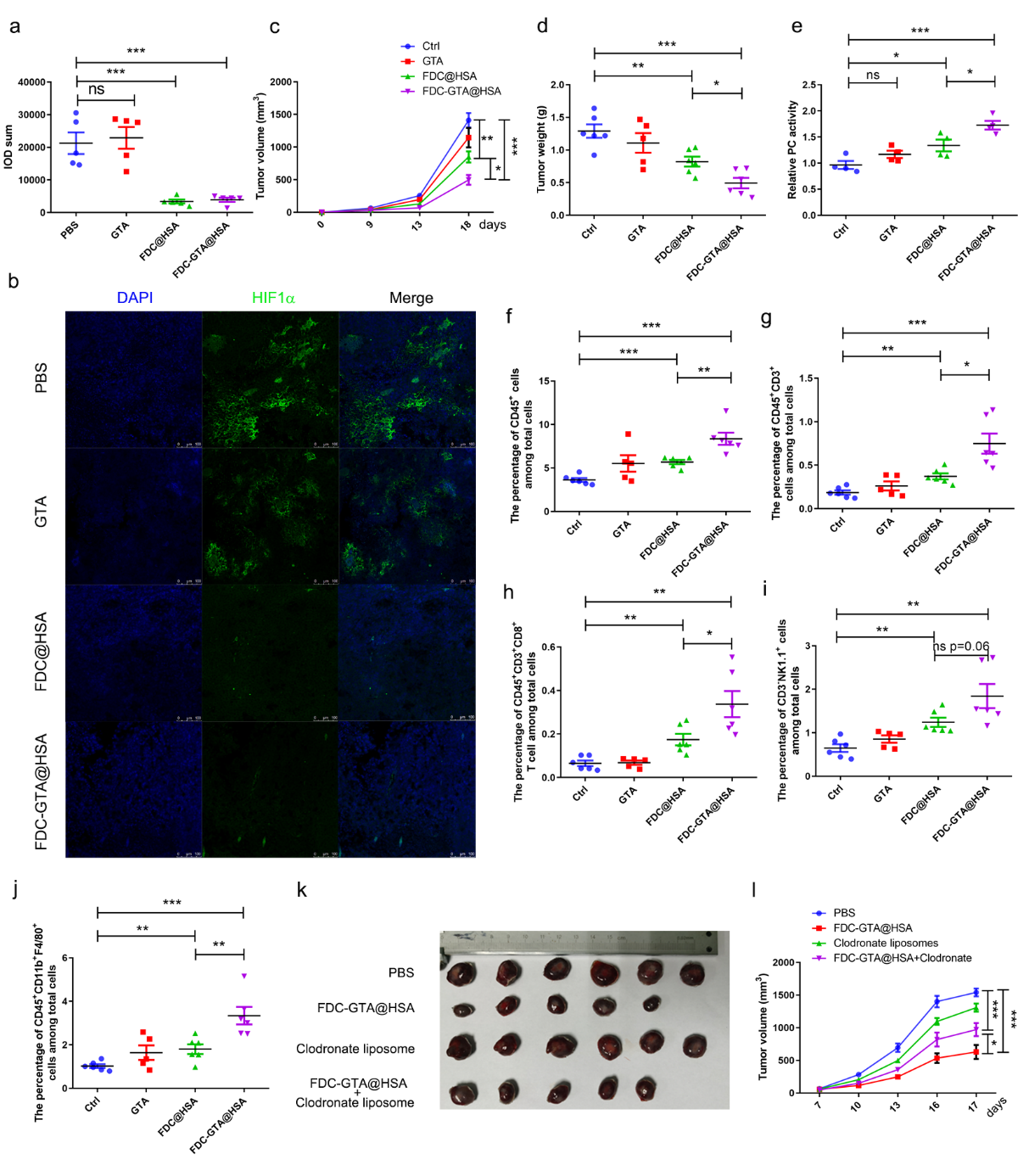


**Fig. S10** FDC-GTA@HSA treatment enhances anti-tumor immunity in vivo. **(a, b)** Statistics (a) and representative images (b) of tumor slices stained with HIF1α. Scale bar, 100 μm. **(c-e)** Tumor growth (c), tumor weight (e), PCB activity (e) in TAMs of indicated groups of mice. **(f-j)** Flow cytometric analysis of CD45^+^ immune cells (f), CD45^+^ CD3^+^ T cells (g), CD45^+^ CD3^+^ CD8^+^ cytotoxic T cells (h), CD45^+^ NK1.1^+^ NK cells (i), CD45^+^ CD11b^+^ F4/80^+^ macrophages (j), among total cells in tumor digests from indicated groups. **(k)** The size of tumors from mice treated with PBS, FDC@HSA, Clodronate liposome or FDC-GTA@HSA+ Clodronate liposome. **(l)** Growth curves of tumors from indicated groups. Data represent as mean±SEM. P<0.05, *; P<0.01, **; P<0.001, *** by Student’s t test. Experiments were repeated at least twice to observe concordant statistical significance.

Table S1.

The top 12 cellular proteins that potentially interacted with PCB

| **Swiss-Prot**  **accession** | **Identified Proteins** | **Score** | **No. of**  **Unique Peptides** | **Molecular weight (KD)** |
| --- | --- | --- | --- | --- |
| **Band1** | | | | |
| Q91Z83 Q02566  G3UW82  A2AQP0  P13542  E9Q390  E9Q7E2 | MYH6 MYH7  MYH2  MYH7b  MYH8  Myof  Arid2 | 323.31  253.77  19.32  12.79  9.56  5.99 | 31  10  2  2  1  1 | 222.88  223.22  221.49  222.7  233.32  195.99 |
| **Band2** | | | | |
| P54071  Q8BWT1  Q9CZU6  P50247  P62334  P5117 | IDH2  Acca2  CS1  Ahcy  Psmc6  Acad1 | 56.95  29.38  27.18  22.46  20.29  13.85 | 3  1  2  2  1  2 | 50.90  41.82  51.73  47.68  44.17  47.89 |
